# A Case Report of Idiopathic Acquired Hemophilia Type A

**DOI:** 10.7759/cureus.38634

**Published:** 2023-05-06

**Authors:** Liz A Amu-Hernández, Cristina Marzo-Alonso, Albert Tugues-Peiró, Eva P Vicente-Pascual, Paula Monteagudo-Aguilar

**Affiliations:** 1 Hematology and Hemostasis, Arnau de Vilanova University Hospital, Lleida, ESP; 2 Thrombosis and Hemostasis Unit, Arnau de Vilanova University Hospital, Lleida, ESP; 3 Internal Medicine, Arnau de Vilanova University Hospital, Lleida, ESP

**Keywords:** coagulation, auto immune, bleeding disorders, novoseven, feiba, autoantibodies, factor viii inhibitor, factor viii, acquired hemophilia

## Abstract

Acquired hemophilia A (AHA) is a rare hemorrhagic coagulopathy caused by the presence of autoantibodies that inhibit the activity of factor VIII (FVIII). Its diagnosis requires a high index of suspicion. It should be suspected in the presence of extensive hematomas or intense mucosal bleeding in patients with no history of previous trauma or hemorrhagic symptoms.

We present two clinical cases of AHA, with different presentations and therapeutic management based on immunosuppression and hemostatic control through bypass agents such as activated recombinant FVII (rFVIIa; Novoseven®) and activated prothrombin complex concentrate (aPCC; Feiba®). The first case was an idiopathic AHA that presented with extensive subcutaneous hematomas with inhibitor titer >40 Bethesda units/ml (BU/mL), prolonged activated partial thromboplastin time (aPTT), and FVIII of 0.8%. In contrast, the second case involved a patient with a history of autoimmune disease, who presented with epistaxis and inhibitor titer of 10.8 BU/ml and FVIII of 5.3%.

## Introduction

Acquired hemophilia (AH), unlike hereditary hemophilia, is a rare hemorrhagic coagulopathy, with a high mortality risk due to underdiagnosis and the consequent delayed initiation of treatment [1]. The incidence of AH is estimated to be between 1-1.5 cases per million population per year [2]. Most of the cases occur in patients above 65 years old almost half of them have an autoimmune disorder or malignancy, but there is also a peak in incidence in pregnant women, especially post-partum [1-5].

AH is caused by autoantibodies that inhibit coagulation factors, with factor VIII (FVIII) being the most affected, leading to acquired hemophilia A (AHA) [1-5]. Coagulation factor inhibitors act through various pathways: either by inhibiting the coagulation activity of the affected coagulation factor, accelerating its catabolism, or clearance. However, the severity of bleeding is not correlated with inhibitor titers [1,3-7]. Autoantibody titers >5 Bethesda units/mL (BU/mL) are considered elevated [1].

The appearance of these coagulation inhibitors seems to be the result of a combination of genetic and environmental factors that alter immune tolerance, such as autoimmune processes, neoplasms, drugs, and post-partum. Up to 10% of AHA cases are associated with solid or hematologic tumors. However, approximately 50% of cases are of idiopathic etiology [1-7]. The most frequent presentation of AH, unlike congenital hemophilia A, is subcutaneous hematoma (>80%), followed by muscle hematoma (>40%) and gastrointestinal bleeding (20%). Other less frequent forms are genitourinary, retroperitoneal, or other bleeding, accounting for <10%. Intracranial hemorrhage is rare but fatal [1-7].

In this report, we present two cases of AHA with different clinical presentations and therapeutic management based on immunosuppression and hemostatic control according to the recent literature.

## Case presentation

Case 1

**Figure 1:**
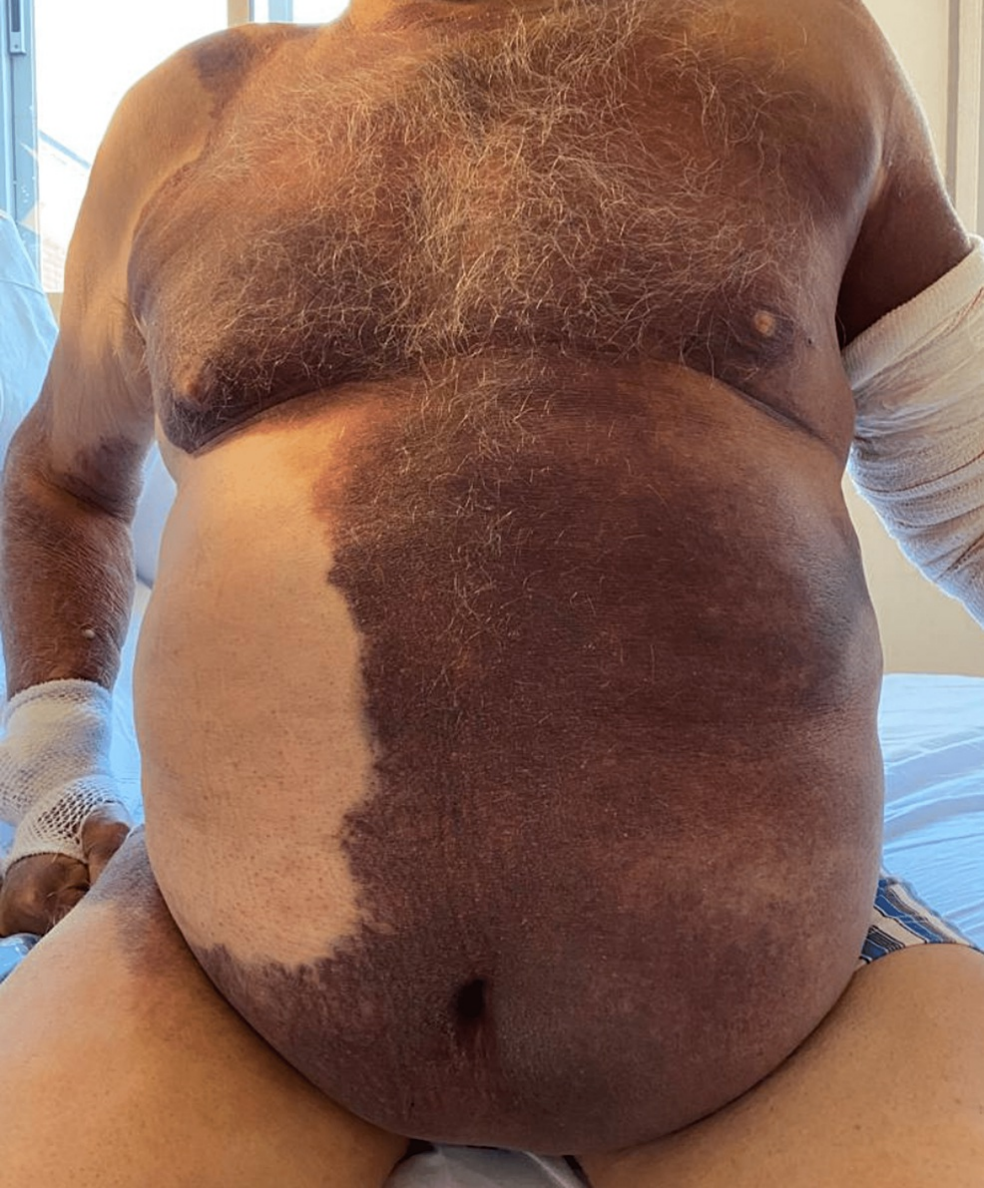
Clinical presentation

A 72-year-old male, who was obese, hypertensive, and with chronic renal disease, presented with extensive subcutaneous hematomas of two months' evolution; he had no traumatic history or history of previous bleeding. His condition had worsened when corticotherapy (prednisone 30 mg per day), which he had been taking for omalgia, was suspended without tapering. Following venoclysis in the left upper extremity, an extensive traumatic hematoma under tension appeared in this extremity, painful on palpation and with paresthesia at the distal level of the joint (Figure 1), which was deemed a significant complication. Laboratory testing showed isolated prolongation of the activated partial thromboplastin time (aPTT) of 4.13 ratio (normal range: 0.8-1.2 ratio), with a normal prothrombin time (PT), normal platelets count, and negative lupus anticoagulant. After confirming that aPTT did not correct to the normal range by performing the mixing test with normal plasma, coagulation factors were identified, with FVIII activity accounting for 0.8%. The titers of inhibitory autoantibodies against FVIII were >40 BU/ml, which led to the diagnosis of AHA.

Immunosuppressive treatment was initiated with intravenous methylprednisolone pulses of 125 mg during the first 72 hours, continuing with doses of methylprednisolone (1 mg/kg/day) that were maintained throughout hospitalization with a progressive decrease at the time of discharge. Due to the absence of response [titers of inhibitory autoantibodies >40 BU/mL and with FVIII activity remaining unchanged (FVIII activity: 0.8%)], other immunosuppressants were added to his regimen [mycophenolate mofetil (MFM) 1 g/12 hours], which was suspended on the sixth day due to persistently high titers of the inhibitor, and bitherapy with cyclophosphamide (CFM) and monoclonal antibody CD20 rituximab (RTX) were added. RTX was administered once a week for three weeks. In addition, intravenous immunoglobulins (IVIG) 35 g were administered for five days.

For hemostatic management, tranexamic acid and activated recombinant factor VII (rFVIIa; Novoseven®) were administered. Novoseven® was discontinued after 10 days of treatment with the achievement of hemoglobin (Hb) and clinical stability. However, after 48 hours, we had to reintroduce Novoseven® due to the appearance of a new spontaneous hematoma and hemodynamic instability. A CT scan of the pelvis showed extensive hematoma formation in the left gluteal region. At the beginning of week four, Novoseven® was replaced with activated prothrombin complex concentrate (aPCC; Feiba®), which led to the control of the bleeding. At week seven, the patient was discharged after he attained clinical stability and showed FVIII inhibitor titer levels of <2 BU/ml. Intensive transfusion support was provided during the first three weeks, and 18 packs of red blood cells were transfused.

During admission, the patient had been screened for autoimmune processes, infections, and neoplasms with negative results. After his hospital discharge and exhaustive follow-up, the patient has not experienced any relapses or complications in the last 12 months.

Case 2 

The second case involved a 73-year-old woman who had been diagnosed with vitiligo and recently started on anticoagulation with warfarin for pulmonary thromboembolism. Two weeks after the initiation of warfarin, the patient experienced an episode of epistaxis, which could not be controlled with nasal packing. Initially, it was associated with anticoagulant treatment, but it persisted even after anticoagulants were withdrawn. The hemostasis study showed normal PT, normal platelets count, and negative lupus anticoagulant; however, a prolonged aPTT ratio was found (aPTT: 3.9 ratio). AHA was confirmed based on low FVIII activity (FVIII: 5%) and high titer of FVIII inhibitor (10.8 BU/ml). After the initiation of immunosuppression with corticosteroids (1 mg/kg/day) and bleeding control with aPCC bypass agent (Feiba®) and tranexamic acid, the patient achieved clinical and analytical stabilization at the beginning of the second week of treatment. She did not require transfusion support (Hb: 11.8 g/dL). The patient was screened for infections, neoplasms, and other autoimmune processes with negative results. At the time of discharge, corticosteroid treatment was maintained in tapering, with no relapses during follow-up.

CT pulmonary angiography and bilateral Doppler lower limb ultrasound were performed, and no pulmonary embolism or deep vein thrombosis was detected. Based on the above findings, we decided to discontinue anticoagulation with the aim to reduce the risk of recurrent and life-threatening bleeding associated with AHA.

## Discussion

AH is a rare hemorrhagic coagulopathy with a high risk of mortality, and prompt diagnosis is required for a good prognosis for these patients. AH should be considered in individuals who present with spontaneous bleeding with no history of trauma or history of previous bleeding, with a normal PT, normal platelets count, and an isolated prolongation of the aPTT in the laboratory tests [1,5-9]. The presence of lupus anticoagulant can confuse the diagnosis of AHA, due to the prolongation of the aPTT, but it will never present with bleeding phenomena as in AH [1]. In both cases reported here, the lupus anticoagulant was negative. Regarding the etiology, although it is idiopathic in most of the reported cases, clinicians should nevertheless maintain a high index of suspicion for AH, especially in older individuals with known autoimmune diseases. Also, as in the first case presented, in patients on corticosteroid treatment with poor adherence to therapy, the suspension of corticosteroids without tapering has been identified as a trigger in the exacerbation of AH [7].

The high risk of mortality due to bleeding in AH is associated with the presence of the inhibitor autoantibody of the coagulation factor; however, the severity of bleeding does not depend on the inhibitor titers [1,3-7]. The poor evolution of AHA has been associated with the presence of high titers of inhibitor autoantibody, the low initial activity of coagulation FVIII, immunoglobulin A (IgA) autoantibody against FVIII, the coexistence of malignancy, and low World Health Organization performance status scale [5,6,10,11]. Therefore, initial treatment usually involves the control of bleeding with bypass agents and immunosuppression to eliminate the clotting factor inhibitor and improve survival. Although rare, the inhibitor can disappear spontaneously, especially in cases associated with drugs and post-partum [1-5]. However, the 2020 AH guidelines recommend providing corticosteroid therapy (1 mg/kg/day) for three to four weeks in all cases [4,10].

Once remission has been achieved, given the high risk of bleeding, it is not advisable to suspend immunosuppressive treatment immediately. Corticosteroids should be maintained for four to six weeks with appropriate tapering. In cases where FVIII is <1% and inhibitor quantification is >20 BU/ml despite corticosteroid therapy, other immunosuppressants, such as CFM or RTX, should be added [1-5,11]. In the second case reported, the patient had inhibitor titers of 10.8 BU/ml with FVIII of 5.3%, achieving stabilization with immunosuppression based on corticotherapy alone. High inhibitor titers are very likely to require the administration of triple therapy (corticosteroids, CFM, and RTX) from the beginning [1]. Other options, such as the administration of IVIG, are not part of the initial management of AH [1-5]. The goal of immunosuppressive therapy is to achieve an inhibitor titer <0.6 BU/ml and FVIII >50% [5,8]. However, a target of FVIII activity levels between 30-50% is sufficient to achieve hemostasis [1].

These patients require close follow-up due to the complications associated with immunosuppression, as there is a risk of sepsis of up to 33%, and on the other hand, the inhibitor must be followed up, although the frequency has not been well established [1]. The second case presented was admitted to the ICU for sepsis of respiratory origin in the context of corticosteroid tapering. Some authors recommend weekly monitoring until the inhibitor is eradicated, and continue with monthly monitoring during the first half of the year due to the high risk of mortality of approximately 50% in this period. Afterward, follow-up can be quarterly/semi-annual based on clinical assessment [1,10,11]. In both cases presented, no relapses occurred during follow-up.

Regarding hemostatic treatment, the severity of bleeding must be considered; 70% of clinical presentations involve severe bleeding, which is variable and does not correlate with coagulation factor levels or inhibitor titers [1,3-7]. Therapeutic strategies for the control of bleeding are based on the use of bypassing agents such as rFVIIa (Novoseven®) and aPCC (Feiba®). Desmopressin and porcine recombinant FVIII are also available but are rarely used due to the superiority of bypass agents [1-5,7,8]. Concerning the differences between the two bypass agents, Feiba® and Novosen®, there have been no trials comparing their efficacy; it seems that both are equally efficacious, and their use is based on clinical experience, the clinical response of the patient, and the costs of use [1,2-5,8,10]. In cases where the first-line bypass agent fails, it is recommended to use the other bypass agent, avoiding the simultaneous use of both agents, which is associated with the high risk of arterial and venous thrombosis, particularly in patients with comorbidities (cardiovascular disease, cancer, and pregnancy) [9]. In our first case, we achieved hemostasis control when the aPCC (Feiba®) was initiated as the second-line bypass agent. Based on this clinical experience, we decided to use aPCC (Feiba®) as the first-line agent in the second case, which led to a good response. Although bypass agents are effective for hemostatic management, they have the disadvantages of high cost, frequent IV infusions, and thromboembolic complications.

Recent case reports suggest that emicizumab (Helimbra®) can reduce the risk of bleeding and the requirements for hemostatic therapy in AHA patients as it does in hereditary hemophilia A. Emicizumab (Helimbra®) is a humanized bispecific monoclonal antibody whose mechanism of action mimics the action of FVIII, binds factors IX and X forming the complex, and thereby activates the coagulation cascade without the need for FVIII. Due to its mechanism of action and subcutaneous route of administration, it is associated with a lower risk of complications than bypass agents. For this reason, emicizumab (Helimbra®) is postulated as the best cost-effective alternative in the hemostatic management of AHA compared to bypass agents [10,11]. However, there are no clinical trials available involving these types of patients.

## Conclusions

AH is an exceedingly rare entity. However, it is associated with a high risk of mortality, not only due to the risk of bleeding associated with the presence of autoantibodies that inhibit coagulation factors but also the time it takes to diagnose it and the associated delay in initiating treatment. AH should be suspected in patients with spontaneous bleeding with no history of trauma or history of previous bleeding, and who, at the analytical level, present normal platelets count and normal PT with prolonged aPTT that does not correct with the mixing test. It is advisable to perform a coagulation study in the setting of bleeding episodes of unknown causes in adulthood, and, above all, a high index of suspicion is required if, in this context, there is a diagnosis of autoimmune disease or erratic immunosuppressive treatment as it can act as a trigger for AH.

Once the entity is recognized, it is important to rapidly initiate immunosuppressive treatment to eradicate the clotting factor inhibitor and to commence hemostatic treatment. Likewise, screening for AHA-triggering factors should be performed, although more than half of the cases are idiopathic in etiology. As it is a rare pathology, there is scarce information on the management of AH in the literature. Hence, there is a need for further research on different lines of treatment and follow-up of this patient population.
